# Inducible Endothelial *Gch1* Deletion Reveals Rapid, Sex-Specific Effects on Blood Pressure and Pregnancy Outcomes

**DOI:** 10.1161/HYPERTENSIONAHA.125.25058

**Published:** 2025-10-01

**Authors:** Surawee Chuaiphichai, Desson Au-Yeung, Christopher A.R. Whiteman, Sarah L. Cook, Eileen McNeill, Gillian Douglas, Keith M. Channon

**Affiliations:** Division of Cardiovascular Medicine, British Heart Foundation Centre of Research Excellence, Radcliffe Department of Medicine, University of Oxford, United Kingdom.

**Keywords:** animals, blood pressure, endothelial cells, fetal development, hypertension, pregnancy

## Abstract

**BACKGROUND:**

Tetrahydrobiopterin (BH4) is an essential cofactor for endothelial nitric oxide synthase. Constitutive endothelial BH4 deficiency leads to mild hypertension and vascular dysfunction that are partly compensated by alternative endothelium-derived vasodilators. Accordingly, we generated a novel VE-Cadherin-CreERT2 (VE-Cad-Cre) mouse to evaluate the impact of inducing endothelial-specific BH4 deficiency in adult animals, without the potential mitigating effects of developmental or other adaptive mechanisms.

**METHODS:**

Endothelial *Gch1* deletion and BH4 deficiency were induced by tamoxifen administration to *Gch1*^*fl/fl*^VE-Cad-Cre male and female adult mice. In female mice, endothelial BH4 deficiency was also induced immediately before pregnancy. The effects of inducible *Gch1* deletion were determined on BH4 levels, vascular function, blood pressure, and fetal development during pregnancy.

**RESULTS:**

Male and female *Gch1*^*fl/fl*^VE-Cad-Cre mice had normal blood pressure. However, tamoxifen treatment of male *Gch1*^*fl/fl*^VE-Cad-Cre mice caused progressive hypertension with impaired nitric oxide synthase-mediated vasodilation. Tamoxifen treatment of female *Gch1*^*fl/fl*^VE-Cad-Cre mice led to nonprogressive hypertension that was exacerbated by pregnancy, leading to impaired uteroplacental vascular function and fetal growth restriction.

**CONCLUSIONS:**

Induction of endothelial cell BH4 deficiency reveals rapid, sex-specific requirements for endothelial cell BH4 in vascular function and blood pressure, and the cardiovascular response to pregnancy. These changes are more striking than those reported for constitutive endothelial cell BH4 deficiency, suggesting a role for developmental or other adaptive effects that fail to mitigate the effects of inducible endothelial cell BH4 deficiency. Targeting endothelial BH4 bioavailability may offer therapeutic strategies for acquired hypertensive disorders and fetal growth restriction.

**NOVELTY AND RELEVANCE**

**What Is New?**

This study is the first to use a tamoxifen-inducible VE-Cadherin-CreERT2 model to selectively delete *Gch1* in adult endothelial cells, bypassing developmental and hematopoietic confounders seen in constitutive models.

Inducible endothelial tetrahydrobiopterin (BH4) deficiency results in a much more severe and sex-specific hypertensive phenotype than previously reported, with progressive hypertension in males and pregnancy-induced hypertension with fetal growth restriction in females.

Unlike constitutive models, this inducible approach reveals a lack of compensatory mechanisms such as endothelial nitric oxide synthase upregulation or hydrogen peroxide-mediated vasodilation, highlighting the critical role of endothelial BH4 in adult vascular homeostasis.

**What Is Relevant?**

This model more accurately mimics adult-onset endothelial dysfunction and acquired vascular disease by inducing BH4 deficiency in mature endothelium.

The sex-specific responses uncovered, particularly the progressive hypertension in males and gestational hypertension in females, offer new insights into sex differences in cardiovascular regulation and risk.

The findings highlight the importance of endothelial BH4 availability not only in systemic blood pressure regulation but also in supporting uteroplacental function during pregnancy.

**Clinical/Pathophysiological Implications?**

Endothelial BH4 deficiency is implicated in essential hypertension and preeclampsia; this model provides a clinically relevant system to study these conditions.

These results suggest that therapeutic strategies aimed at restoring endothelial BH4 could have a significant benefit in acquired vascular diseases and hypertensive pregnancy syndromes.

Targeting BH4 bioavailability may offer precision medicine approaches for sex-specific cardiovascular and pregnancy-related complications.

Tetrahydrobiopterin (BH4) is a crucial enzymatic cofactor for the activity of nitric oxide synthases (NOSs), which play a key role in regulating vascular homeostasis, endothelial function, and blood pressure (BP).^1,2^ The biosynthesis of BH4 primarily depends on the enzyme GTPCH (GTP cyclohydrolase 1), encoded by *Gch1*, which catalyzes the rate-limiting step of the BH4 biosynthesis. The requirement for endothelial BH4 is particularly important in pregnancy, where increased cardiac output, decreased systemic vascular resistance, and uteroplacental vascular remodeling are crucial to support the growing fetus.^3–5^ Constitutive endothelial cell BH4 deficiency in female mice results in pregnancy-induced hypertension, placental insufficiency, and fetal growth restriction.^3,4^ Given the pivotal role of endothelial BH4 in vascular homeostasis, understanding its regulation and function in the endothelium is crucial for developing targeted therapies for hypertension and related disorders.

Genetic mouse models have made important contributions to the understanding the role of BH4 in endothelial function and vascular homeostasis. Deletion of *Gch1*, which prevents de novo BH4 biosynthesis, is embryonically lethal in global *Gch1* knockout mice,^6^ but cell-specific *Gch1* knockouts are viable and have selective loss of BH4 in *Gch1*-deleted cells, demonstrating a cell-autonomous requirement for *Gch1* and BH4 that is not rescued by otherwise normal systemic BH4 levels.^7^ Furthermore, systemic BH4 supplementation does not necessarily rescue the effects of cell-specific BH4 deficiency,^6^ indicating important roles for BH4 distribution and transport. Prior studies using Tie2-cre-mediated *Gch1* knockout mice revealed a critical requirement for endothelial cell *Gch1* and BH4.^7,8^ These animals show only a modest elevation in BP (≈7 mm Hg) due to maintained endothelial-dependent relaxation, mediated by other mediators such as hydrogen peroxide. Other developmental or adaptive mechanisms may also be important, as Tie2cre-mediated *Gch1* deletion has developmental effects on hematopoietic and embryonic tissues, reflecting the transient expression of *Tie2* in other lineages during embryonic development.^7,9,10^ Thus, constitutive *Gch1* deletion only reveals the effects of long-term BH4 deficiency, which may implicate not just the immediate biochemical effects of BH4 deficiency, but also the potential long-term consequences of developmental or other adaptive changes, limiting the applicability to acquired vascular dysfunction states. To overcome these limitations, we developed a novel inducible model to selectively delete *Gch1* in endothelial cells (ECs) in adult mice. This approach enables the investigation of adult-onset endothelial cell-specific BH4 deficiency, avoiding the potential confounding effects of developmental or adaptive mechanisms. We used this model to elucidate both the immediate and long-term effects of inducing endothelial BH4 deficiency on BP and vascular function, in both male and female mice, and on pregnancy outcomes in female mice.

## Methods

### Data Availability

The authors declare that all supporting data are available within the article and in the Supplemental Material.

### Generation of Endothelial Cell-Targeted *Gch1* Knockout Mice

To generate mice with a tamoxifen-inducible, endothelial-specific deletion of *Gch1* (*Gch1*^*fl/fl*^VE-Cadherin-CreERT2 [VE-Cad-Cre]), *Gch1*^*fl/fl*^ mice were first crossed with Cdh5-Cre^ERT2^ (PAC) mice (VE-Cad-Cre) mice,^11^ (provided by Dr R. Adams) to obtain *Gch1*^*fl/+*^VE-Cad-Cre mice. These heterozygous mice were subsequently crossed with *Gch1*^*fl/fl*^ mice^7^ to generate *Gch1*^*fl/fl*^VE-Cad-Cre mice and their respective littermate controls. All mice had been back-crossed on to a C57bl/6J strain background. Mice were housed in individually ventilated cages (4–6 mice per cage, mixed genotypes) under specific pathogen-free conditions, with a controlled 12-hour light/dark cycle and a regulated ambient temperature (20–22 °C). All mice were fed a standard chow diet (Teklad global 16% protein diet, Harlan Laboratories) and provided water ad libitum. For experimental procedures, adult *Gch1*^*fl/fl*^VE-Cad-Cre mice and their *Gch1*^*fl/fl*^ littermates (hereafter referred to as wild-type [WT]) on a pure (>10 generations) C57BL/6J background were bred in-house and used at 10 to 16 weeks of age. The generation and characterization of the knockout model were conducted in accordance with the Animal (Scientific Procedures) Act 1986. In all experimental studies, mice were randomized, and researchers conducting gavage, tissue collection and analysis were blinded to genotype. Adult male and female mice homozygous for floxed *Gch1* and carrying an endothelial-specific Cre-ERT2 transgene (ie, VE-Cad-Cre) were treated via oral gavage with tamoxifen (2 mg per dose; Sigma, 100 µL of 20 mg/ml; dissolved in peanut oil) once daily for 3 consecutive days to induce endothelial-specific deletion of *Gch1*. All procedures were reviewed and approved by the Clinical Medicine Animal Care and Ethical Review Body and conducted under project license PPL P0C27F69A.

Genotyping of mice was performed using polymerase chain reaction (PCR) analysis of genomic DNA extracted from ear biopsies. For *Gch1*^*fl/fl*^ genotyping, PCR was performed using the following primers: *Gch1*^*fl/fl*^-Fw 5′-GTC CTT GGT CTC AGT AAA CTT GCC AGG-3′, *Gch1*^*fl/fl*^-Rv 5′-GCC CAG CCA AGG ATA GAT GCA G-3′. The *Gch1* floxed allele produced a 1030 bp fragment. For Cre genotyping, PCR was performed using the following primers: Cre Fw 5′-GCA TAA CCA GTG AAA CAG CAT TGC TG-3′. Cre Rv 5′-GGA CAT GTT CAG GGA TCG CCA GGC G-3′. The Cre allele produced a 280 bp fragment.

### Genomic DNA Production and Excision PCR

Genomic DNA for detection of the excised allele was produced using the QIAamp kit (Qiagen). The floxed and excised allele were detected using the following primers: 5′GTC CTT GGT CTC AGT AAA CTT GCC AGG3′, 5′GCC CAG CCA AGG ATA GAT GCA G3′, and 5′GCT CAT CCC CCA CAC TTG TCT T3′. The *Gch1* floxed allele yields a 1030 bp and the excised allele a production of 1392 bp.

### Isolation of Murine ECs

Primary lung/heart ECs were isolated using MACS beads (Miltenyi Biotec), as described previously.^12,^^13^ Briefly, mice were culled by overdose of inhaled isoflurane. Lungs/heart were harvested and digested in DMEM containing 0.18 unit/mL Liberase (Roche) and 0.1 mg/mL Dnase I (Roche) for 1 hour at 37 °C. The digested tissue was filtered through 100 µm and 70 µm cell strainers. The cell suspension was then incubated with rat anti-CD31 antibody (BD Pharmingen) for 15 minutes at 4 °C then with anti-rat secondary antibody coated immune magnetic beads for further 15 minutes at 4 °C. Bead-bound ECs were selected using a magnetic column. In all experiments, ECs were collected directly from the magnetic column and used for biochemical analyses.

### Quantification of Intracellular BH4, BH2, and Biopterin by HPLC

Intracellular concentrations of tetrahydrobiopterin (BH4), dihydrobiopterin (BH2), and biopterin were measured in mouse tissue or cell pellets using high-performance liquid chromatography (HPLC) with electrochemical and fluorescence detection, following an established protocol.^14,15^ Briefly, cells were subjected to freeze-thaw lysis in ice-cold resuspension buffer containing 50 mmol/L PBS, 1 mmol/L dithiothreitol, and 1 mmol/L ethylenediaminetetraacetic acid at pH 7.4. The lysates were centrifuged at 13 200 rpm for 10 minutes at 4 °C, and the supernatant was carefully removed. To precipitate proteins and stabilize biopterins, an ice-cold acid precipitation reagent (1 mmol/L phosphoric acid, 2 M trichloroacetic acid, 1 mmol/L dithiothreitol) was added to the supernatant. Samples were mixed thoroughly, followed by centrifugation at 13 000 rpm for 5 minutes at 4 °C. The resulting supernatants were transferred to vials and injected into an isocratic HPLC system equipped with sequential electrochemical (Coulochem III, ESA, Inc) and fluorescence (Jasco) detection. Chromatographic separation was achieved using a 250 mm ACE C-18 column (Hichrom) and a mobile phase consisting of 50 mmol/L sodium acetate, 5 mmol/L citric acid, 48 μM ethylenediaminetetraacetic acid, and 160 μM dithiothreitol at pH 5.2 (all ultrapure electrochemical HPLC-grade reagents). The flow rate was set at 1.3 mL/min. BH4 detection was performed using an electrochemical detector with background currents of +500 μA and −50 μA applied to electrochemical cells E1 and E2, respectively. BH2 and biopterin were quantified using a Jasco FP2020 fluorescence detector with an excitation wavelength of 350 nm and an emission wavelength of 450 nm. Concentrations of BH4, BH2, and biopterin were determined by comparison with authentic external standards and normalized to total protein content.

### Vasomotor Function Studies

Vasomotor function in mesenteric arteries and aortas was assessed in WT and *Gch1*^*fl/fl*^VE-Cad-Cre mice, including both male and female cohorts, as well as nonpregnant (age-matched) and pregnant (E18.5) *Gch1*^*fl/fl*^VE-Cad-Cre and WT littermates. Isometric tension studies were performed using a wire myograph (MultiMyograph 610 M, Danish Myo Technology, Denmark). Mice were euthanized via overdose of inhaled isoflurane, and vascular rings were carefully isolated from the mesenteric arcades and thoracic aorta as previously described.^5,8^ Briefly, segments of second-order mesenteric artery and thoracic aortas were carefully dissected free from surrounding fat and connective tissue and cut into a 2-mm long rings. Two 25 µm diameter Gold Tungsten (for mesenteric arteries) and 40 µm diameter stainless steel wires (for aortas) were carefully threaded through the lumen of each arterial ring, ensuring the endothelium remained intact. The wires were then positioned between the mounting support jaws of a 4-chamber myograph chamber containing 5 mL of ice-cold Krebs-Henseleit buffer, composed of (in mmol·L^−^^1^): NaCl 120, KCl 4.7, MgSO_4_ 1.2, KH_2_PO_4_ 1.2, CaCl_2_ 2.5, NaHCO_3_ 25, and glucose 5.5. The buffer was maintained at 37 °C and continuously gassed with 95% O_2_/5% CO_2_. After an initial 30-minute equilibration period, vessels were set to an optimal resting pressure equivalent to 100 mm Hg. Concentration-response contraction curves were generated by cumulatively adding half-log concentrations of U46619 (thromboxane A_2_ receptor agonist) for mesenteric arteries and phenylephrine for aortas. Following contraction, vessels were washed 3× with fresh Krebs-Henseleit buffer, equilibrated for 20 minutes, and contracted to 80% to 90% of maximal tension using U46619 (mesenteric arteries) or phenylephrine (aortas). Endothelium-dependent vasodilation was assessed by applying increasing cumulative concentrations of acetylcholine, with responses expressed as a percentage of the precontracted tension. Endothelium-independent vasorelaxation was tested using the nitric oxide donor sodium nitroprusside (SNP) in the presence of L-NAME (Nω-nitro-l-arginine methyl ester; 100 μM) to inhibit endogenous nitric oxide synthase activity. All drugs were obtained from Sigma Chemical Company.

### Timed Mating

Pregnancy was achieved by mating virgin female *Gch1*^*fl/fl*^VE-Cad-Cre or *Gch1*^*fl/fl*^ (WT) mice, aged between 10 and 16 weeks, with male *Gch1*^*fl/fl*^ mice. To determine gestational timing, vaginal plugs were checked daily in the morning, with the presence of a plug indicating embryonic day 0.5 (E0.5) of gestation. Unless otherwise specified, tissue samples were collected either at the preconception stage (before timed mating) or at embryonic day 18.5 (E18.5), corresponding to late gestation, 1 day before normal term delivery. All samples were processed immediately for experimental analyses.

### BP Measurement by Tail-Cuff Plethysmography

BP in conscious WT and *Gch1*^*fl/fl*^VE-Cad-Cre mice was measured using the Visitech computerized tail-cuff plethysmography system (BP-2000; Visitech). Before data collection, mice underwent a 5-day training period to acclimatize to the procedure, followed by 3 consecutive days of baseline measurements. All experiments were performed between 8:00 am and 12:00 pm to minimize diurnal variations and were conducted in a designated quiet area (22±2 °C). During measurements, the tail of each mouse was passed through the inflatable cuff and positioned on the photoplethysmographic sensor and secured with tape to minimize movement artifacts, with animals placed in individual restrainers on a temperature-controlled platform maintained at ≈35 °C. A total of 20 BP readings were obtained per mouse, with the first 5 readings discarded to reduce variability. The remaining 15 readings were averaged to determine the mean tail-cuff BP for each mouse. Tail-cuff BP was assessed in both male and female mice at baseline (pretamoxifen administration) and at 4, 12, and 24 weeks post-tamoxifen administration. In pregnant females, tail-cuff BP was monitored throughout gestation in *Gch1*^*fl/fl*^VE-Cad-Cre and WT mice at embryonic days E0, E2.5, E5.5, E7.5, E10.5, E12.5, E15.5, E16.5, E17.5, and E18.5.

### Solutions and Drugs

All drugs were obtained from Sigma-Aldrich (Poole, United Kingdom). All drugs were dissolved in distilled water, with the exception of U46619, which was dissolved in dimethyl sulfoxide (DMSO) and then diluted in physiological buffer for experimentation (pH 7.4 at 37 °C), keeping the final DMSO concentration below 1:1000 to avoid vehicle-associated artifacts.

### Statistical Analysis

Data are presented as mean±SEM. Normality was tested using the D’Agostino and Pearson omnibus normality test. Groups were compared using the Mann-Whitney *U* test for nonparametric data or an un-paired Student *t* test for parametric data. When comparing multiple groups, data were analyzed by ANOVA with Newman-Keuls posttest for parametric data or Kruskal-Wallis test with Dunns posttest for nonparametric data. When more than 2 independent variables were present, a 2-way ANOVA with Tukey multiple comparisons test was used. When within subject repeated measurements were present, a repeated measures ANOVA was used. A value of *P*<0.05 was considered statistically significant and *P* values between 0.05 and 0.10 were considered indicative of a trend.

## Results

### Induction of Endothelial Cell-Specific *Gch1* Deletion Leads to BH4 Deficiency in Endothelial-Rich Tissues Without Affecting Nonendothelial Tissues

**Figure 1.**
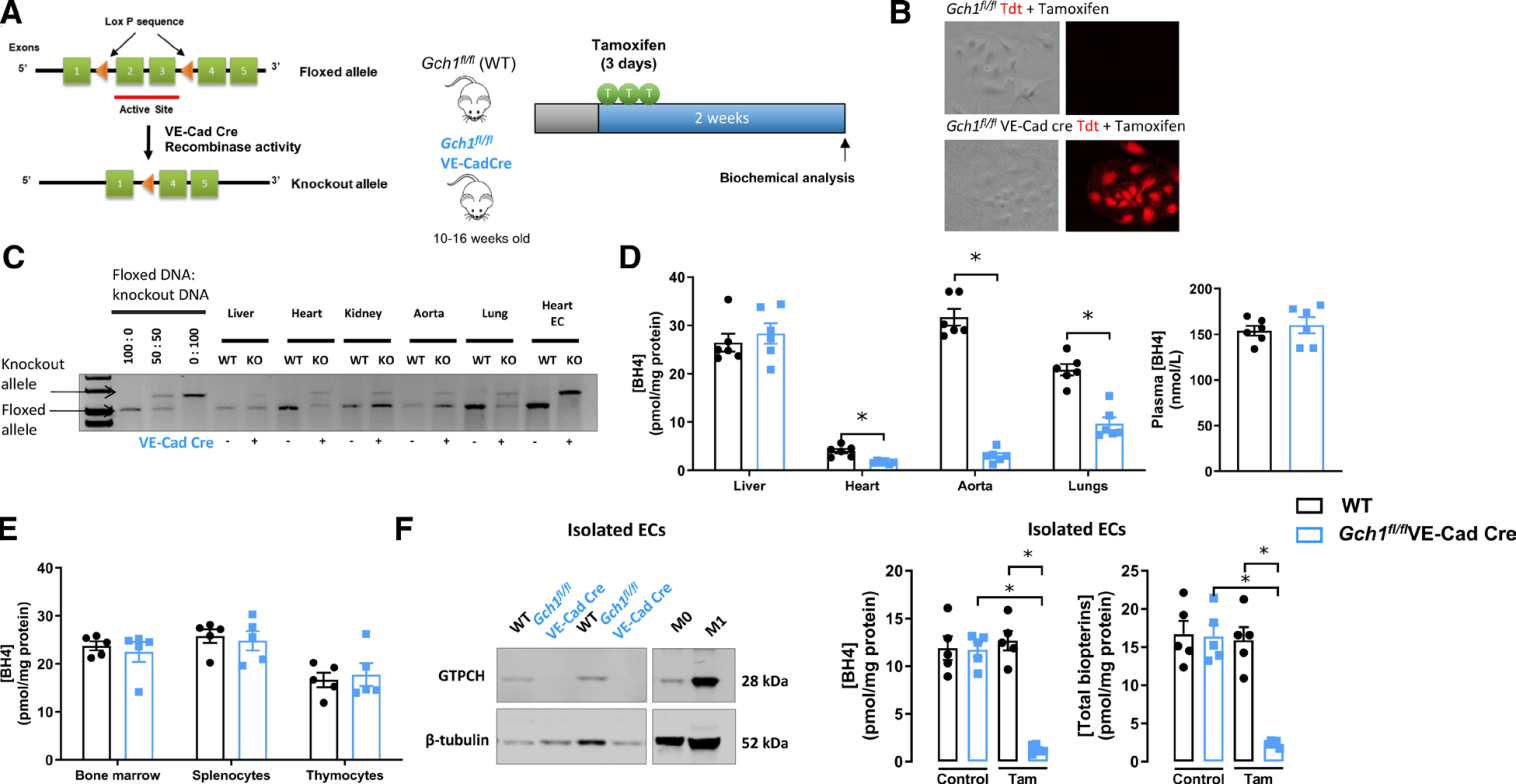
**Induction of endothelial cell-specific Gch1 deletion leads to tetrahydrobiopterin (BH4) deficiency in endothelial-rich tissues without affecting nonendothelial tissues. A**, Schematic representation of the floxed *Gch1* locus, showing the insertion of LoxP sites flanking exons 2 and 3, which encode the active site of GTP cyclohydrolase I (GTPCH; **left**). Experimental protocol outlining the inducible *Gch1* knockout strategy. Adult *Gch1*^*fl/fl*^ (wild-type [WT]) and *Gch1*^*fl/fl*^VE-Cad-Cre littermates were administered tamoxifen (2 mg/day for 3 consecutive days) to activate Cre recombinase (**right**). **B**, Representative images of isolated endothelial cells from WT and *Gch1*^*fl/fl*^VE-Cad-Cre carrying a TdTomato reporter confirm tamoxifen-induced VE-Cad-Cre-mediated recombination in endothelial cells. The presence of TdTomato fluorescence indicates successful Cre-dependent recombination in *Gch1*^*fl/fl*^VE-Cad-Cre mice. **C**, Analysis of VE-Cad-Cre-mediated excision of the LoxP-flanked *Gch1* allele in tissues and primary endothelial cells (ECs) isolated from *Gch1*^*fl/fl*^VE-Cad-Cre and *Gch1*^*fl/fl*^ (WT) mice. The expected 1030 bp product was detected in WT mice. In *Gch1*^*fl/fl*^VE-Cad-Cre mice, the 1392 bp knockout allele was efficiently excised, with excision confirmed in primary ECs. **D** and **E,** Quantification of BH4 levels in tissues, plasma and hematopoietic cells from *Gch1*^*fl/fl*^VE-Cad-Cre and WT mice (*P<0*.05*; n=5–6 per group). **F.** Representative immunoblot analysis of GTPCH (GTP cyclohydrolase 1) protein levels in isolated primary ECs derived from WT and *Gch1*^*fl/fl*^VE-Cad-Cre mice. β-tubulin was used as a loading control (**left**). Stimulated (M1) and unstimulated (M0) wild-type macrophages were used as positive control and controls for GTPCH, respectively. Quantification of BH4 and total biopterin levels in primary ECs from *Gch1*^*fl/fl*^VE-Cad-Cre and WT mice following tamoxifen treatment (2 mg/day for 3 consecutive days) or vehicle treatment. (*P<0*.05*; n=5 animals per group; **right**). Data are presented as mean±SEM.

We generated matched litters of *Gch1*^*fl/fl*^VE-Cad-Cre and *Gch1*^*fl/fl*^ (WT) mice by crossing male *Gch1*^*fl/fl*^VE-Cad-Cre with female *Gch1*^*fl/fl*^ mice (Figure 1A). To induce conditional *Gch1* deletion, mice were orally administered 2 mg of tamoxifen for 3 consecutive days (Figure 1A). Tissues and cells were collected 2 weeks post-tamoxifen administration for biochemical analysis. The body weights of *Gch1*^*fl/fl*^ and *Gch1*^*fl/fl*^VE-Cad-Cre mice were comparable in both males and females before and after tamoxifen administration. Tamoxifen-induced recombination mediated by VE-Cad-Cre in ECs was confirmed using ECs isolated from a TdTomato reporter mouse, in which TdTomato expression is activated only upon Cre-mediated excision of a loxP-flanked STOP cassette (Figure 1B). Genomic PCR confirmed efficient excision of the floxed *Gch1* allele in primary ECs isolated from *Gch1*^*fl/fl*^VE-Cad-Cre mice, but not in ECs from WT mice (Figure 1C). BH4 levels in endothelial cell-rich tissues such as the heart, lung, and aorta were significantly reduced in *Gch1*^*fl/fl*^VE-Cad-Cre mice compared with WT controls (Figure 1D and Figure S1). In contrast, BH4 levels in nonendothelial cell-rich tissues, such as the liver, remained unchanged between WT and *Gch1*^*fl/fl*^VE-Cad-Cre mice. Furthermore, plasma BH4 levels were comparable between the groups (Figure 1D).

Importantly, BH4 levels in hematopoietic cells, including those in bone marrow, as well as immune cells in the spleen and thymus (splenocytes and thymocytes), were not significantly different between WT and *Gch1*^*fl/fl*^VE-Cad-Cre mice, indicating that *Gch1* deletion was restricted to ECs (Figure 1E and Figure S2). To further validate the endothelial specificity of *Gch1* deletion and its impact on BH4 biosynthesis, we isolated primary mouse ECs using immunomagnetic bead selection. Western blot analysis revealed that GTPCH protein was barely detectable in ECs from *Gch1*^*fl/fl*^VE-Cad-Cre mice, whereas it was readily detectable in WT ECs (Figure 1F). No significant differences in P-endothelial NOS (eNOS) Ser1177/eNOS, P-eNOS Thr495/eNOS, or total eNOS/β-tubulin were observed between genotypes (Figure S3). HPLC measurements of biopterins confirmed a marked reduction in BH4 levels in primary ECs isolated from *Gch1*^*fl/fl*^VE-Cad-Cre mice compared with those from WT mice (Figure 1F).

These findings indicate that induction of endothelial-specific *Gch1* deletion in adult mice, using the VE-Cad-CreERT2 system, leads to a selective deficiency of BH4 in ECs, without effects on nonECs or bone marrow-derived cells.

### Induction of Endothelial BH4 Deficiency in Adult Mice Causes Hypertension

**Figure 2.**
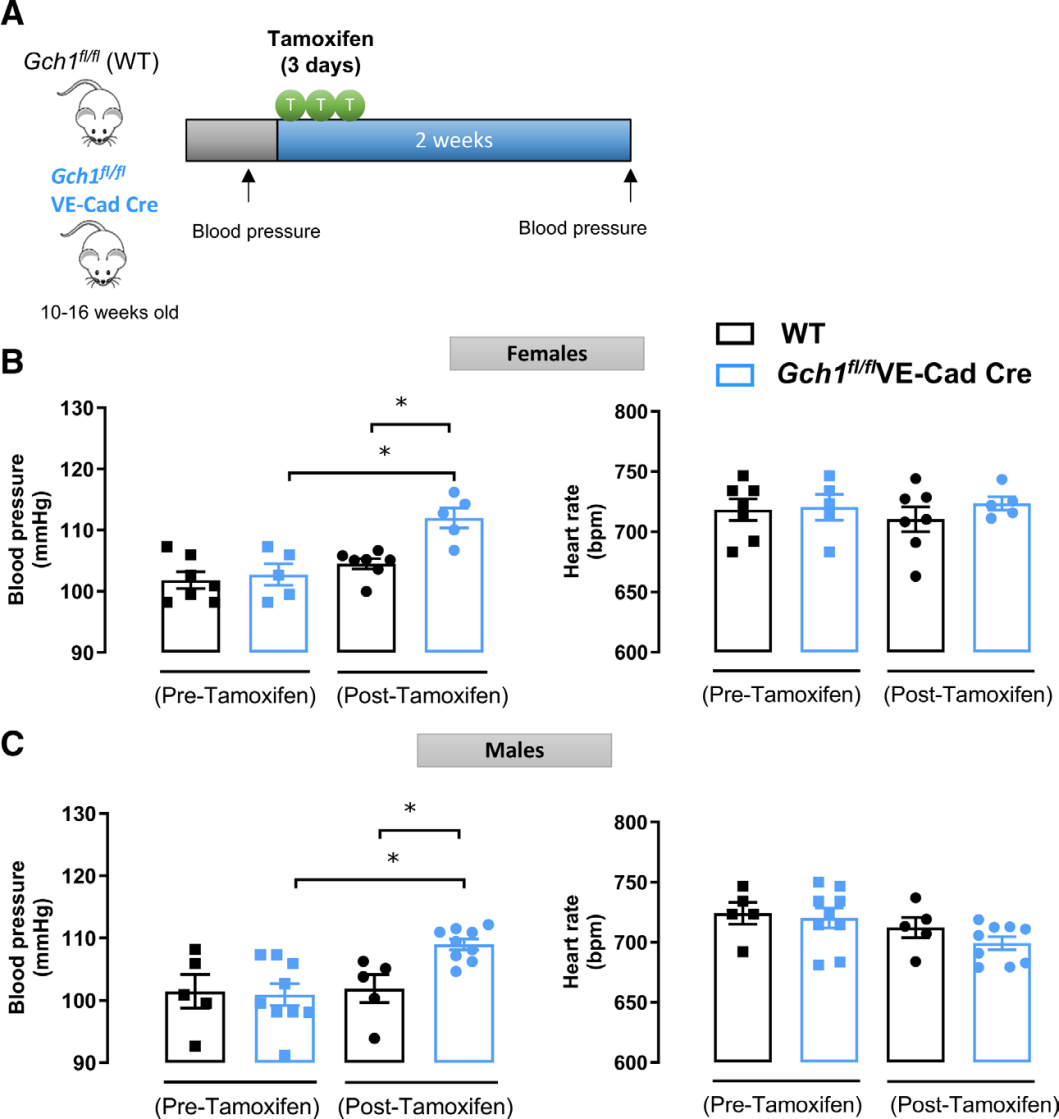
**Induction of endothelial tetrahydrobiopterin (BH4) deficiency in adult mice causes hypertension. A**, Endothelial cell-specific *Gch1* knockout mice were generated through tamoxifen administration (2 mg/day for 3 consecutive days) starting at 10 to 16 weeks of age. Tail-cuff blood pressure (BP) was measured by tail-cuff plethysmography in both (**B**) female and (**C**) male adult *Gch1*^*fl/fl*^ (wild-type [WT]) and *Gch1*^*fl/fl*^VE-Cad-Cre mice, both pre- and 2 weeks post-Cre-mediated excision of *Gch1* in endothelial cells. Each data point represents an individual mouse (*P<0*.05*; n=5–9 animals per group). Data are presented as mean±SEM.

To investigate the effects of induction of endothelial *Gch1*/BH4 deficiency on BP regulation in vivo, BP was measured in conscious WT and *Gch1*^*fl/fl*^VE-Cad-Cre mice using tail-cuff plethysmography. Measurements were conducted in both male and female mice at baseline (pretamoxifen administration) and 2 weeks post-tamoxifen administration (Figure 2A). Before tamoxifen administration, tail-cuff BP was comparable between WT and *Gch1*^*fl/fl*^VE-Cad-Cre mice in both sexes (Figure 2B and 2C). However, 2 weeks following tamoxifen administration, tail-cuff BP was significantly elevated (≈5–10 mm Hg) in both male and female *Gch1*^*fl/fl*^VE-Cad-Cre mice compared with their WT littermate controls (Figure 2B and 2C).

Heart rate remained unchanged between female *Gch1*^*fl/fl*^VE-Cad-Cre and *Gch1*^*fl/fl*^ mice, both before and 2 weeks after tamoxifen administration (Figure 2B). In male *Gch1*^*fl/fl*^VE-Cad-Cre mice, there was a trend toward a reduction in heart rate at 2 weeks post-tamoxifen administration compared with baseline levels (ie, pretamoxifen administration; Figure 2C).

Taken together, these findings indicate that inducible endothelial cell-specific *Gch1* deletion and subsequent BH4 deficiency in adult mice result in a significant increase in BP, within 2 weeks, in both male and female mice, highlighting the critical requirement for endothelial cell BH4 in BP regulation, independent of developmental or other longer term adaptive effects.

### Hypertensive Response to Induction of Endothelial Cell *Gch1* Deletion and BH4 Deficiency is Sex-Specific

**Figure 3.**
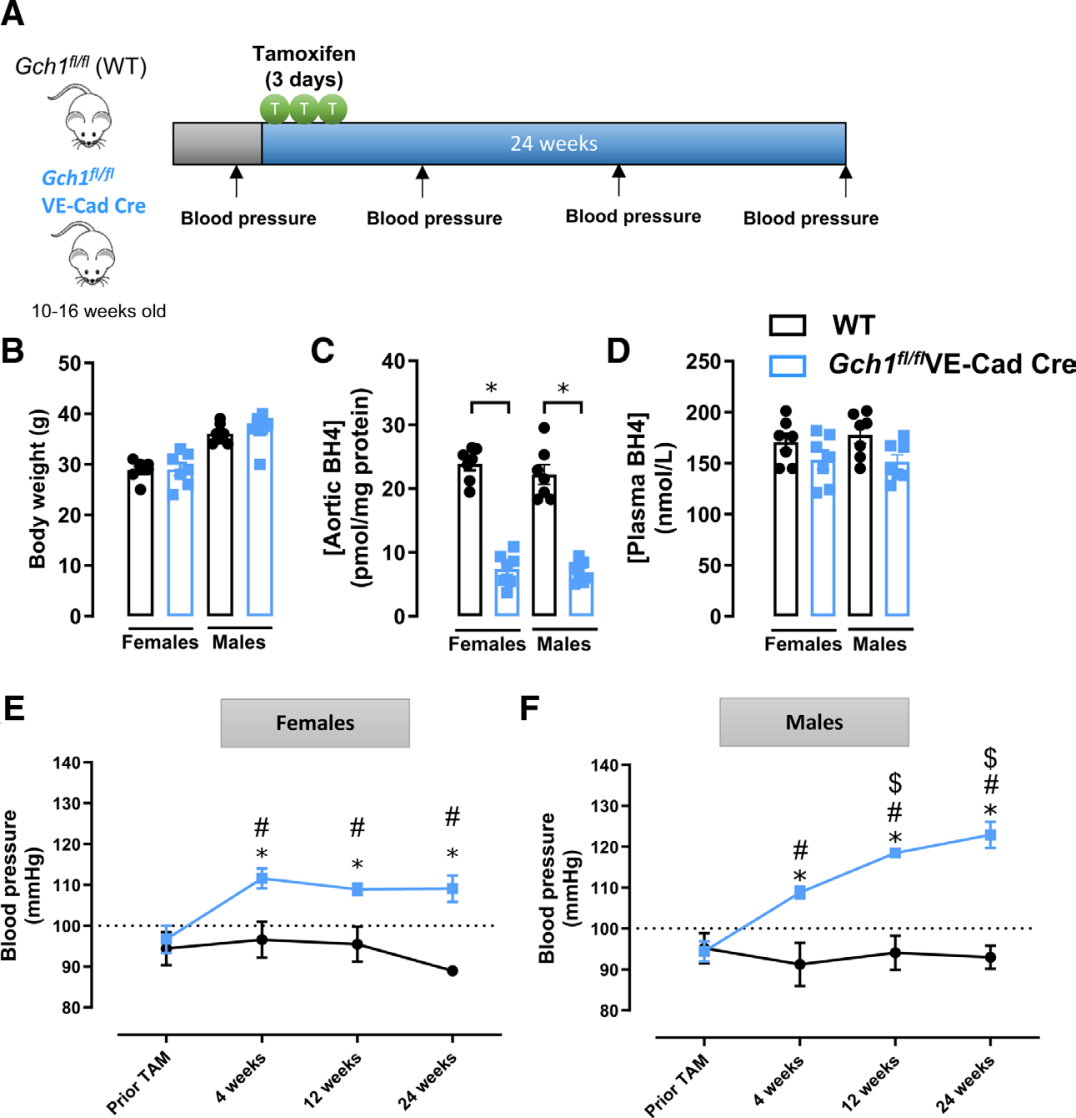
**The hypertensive response to induction of endothelial cell *Gch1* deletion and tetrahydrobiopterin (BH4) deficiency is sex-specific. A**, Endothelial cell-specific *Gch1* knockout mice were generated by tamoxifen administration (2 mg/day for 3 consecutive days) starting at 10 to 16 weeks of age. Blood pressure (BP) was measured by tail-cuff plethysmography in both male and female adult *Gch1*^*fl/fl*^ (wild-type [WT]) and *Gch1*^*fl/fl*^VE-Cad-Cre mice at 4, 12, and 24 weeks post-Cre-mediated *Gch1* deletion in endothelial cells. **B**, Body weight of males and females 24 weeks post-tamoxifen (n=7 animals per group). **C** and **D**, BH4 levels in aorta and plasma from both male and female WT and *Gch1*^*fl/fl*^VE-Cad-Cre at 24 weeks post-tamoxifen respectively (*P<0*.05*; n=7 animals per group). **E**, In female mice, tail-cuff BP was significantly elevated in *Gch1*^*fl/fl*^VE-Cad-Cre mice compared with WT controls as early as 4 weeks post-tamoxifen administration. This elevation in blood pressure was sustained and remained significantly higher at 12 and 24 weeks post-tamoxifen administration (*P<0*.05*; vs WT at the same time point, *#P*<0*.05*; vs Pretamoxifen *Gch1*^*fl/fl*^VE-Cad-Cre; n=7 animals per group). **F**, In male mice, tail-cuff BP was also significantly increased in *Gch1*^*fl/fl*^VE-Cad-Cre mice compared with wild-type controls. Furthermore, there was a progressive increase in BP over time, with BP at 12 and 24 weeks post-tamoxifen administration being significantly higher than at 4 weeks. Each data point represents an individual mouse (*P<0*.05*; vs WT at the same time point, *#P*<0*.05* vs Pretamoxifen *Gch1*^*fl/fl*^VE-Cad-Cre; $*P*<0*.05;* vs *Gch1*^*fl/fl*^VE-Cad-Cre at 4 weeks post-tamoxifen; n=7 animals per group). Data are presented as mean±SEM.

To assess the long-term impact of endothelial *Gch1*/BH4 deficiency on BP, tail-cuff BP was measured in conscious WT and *Gch1*^*fl/fl*^VE-Cad-Cre mice at baseline (pretamoxifen) and 4, 12, and 24 weeks post-tamoxifen (Figure 3A). At 24 weeks, body weight remained comparable between WT and *Gch1*^*fl/fl*^VE-Cad-Cre mice in both sexes (Figure 3B; females: WT: 28.8±1.9 g, *Gch1*^*fl/fl*^VE-Cad-Cre: 29.0±3.2 g; males: WT: 36.1±1.9 g, *Gch1*^*fl/fl*^VE-Cad-Cre: 37.2±2.9 g; Figure 3B). Aortic BH4 levels remained significantly reduced in *Gch1*^*fl/fl*^VE-Cad-Cre mice, consistent with previous findings at 2 weeks, while plasma BH4 levels were unchanged between genotypes (Figure 3D and 3D).

At baseline, tail-cuff BP was similar between groups in both sexes (Figure 3E; females: WT: 94±9 mm Hg versus *Gch1*^*fl/fl*^VE-Cad-Cre: 96±7 mm Hg). However, in females, BP was significantly increased at 4 weeks post-tamoxifen (Figure 3E; WT: 97±9 mm Hg versus *Gch1*^*fl/fl*^VE-Cad-Cre: 111±5 mm Hg) and remained elevated through 12 and 24 weeks (Figure 3E; for 12 weeks: WT: 95±8 mm Hg versus *Gch1*^*fl/fl*^VE-Cad-Cre: 109±3 mm Hg; for 24 weeks: WT: 89±2 mm Hg versus *Gch1*^*fl/fl*^VE-Cad-Cre: 109±7 mm Hg), indicating sustained hypertension. In males, BP was also increased at 4 weeks but continued to rise progressively at 12 and 24 weeks, indicating a progressive hypertensive phenotype over time (Figure 3F; for 12 weeks: WT: 94±9 mm Hg versus *Gch1*^*fl/fl*^VE-Cad-Cre: 119±2 mm Hg; for 24 weeks: WT: 93±6 mm Hg versus *Gch1*^*fl/fl*^VE-Cad-Cre: 123±7 mm Hg).

These findings demonstrate that induction of endothelial cell *Gch1* deletion leads to persistent hypertension due to sustained BH4 deficiency. The hypertensive response is sex-dependent, with male mice exhibiting a progressive increase in BP over time, whereas in female mice the hypertensive response to induction of endothelial BH4 deficiency is more modest and not progressive.

### Induction of Endothelial Cell *Gch1* Deletion Impairs Resistance Vessel Function in a Sex-specific Manner

**Figure 4.**
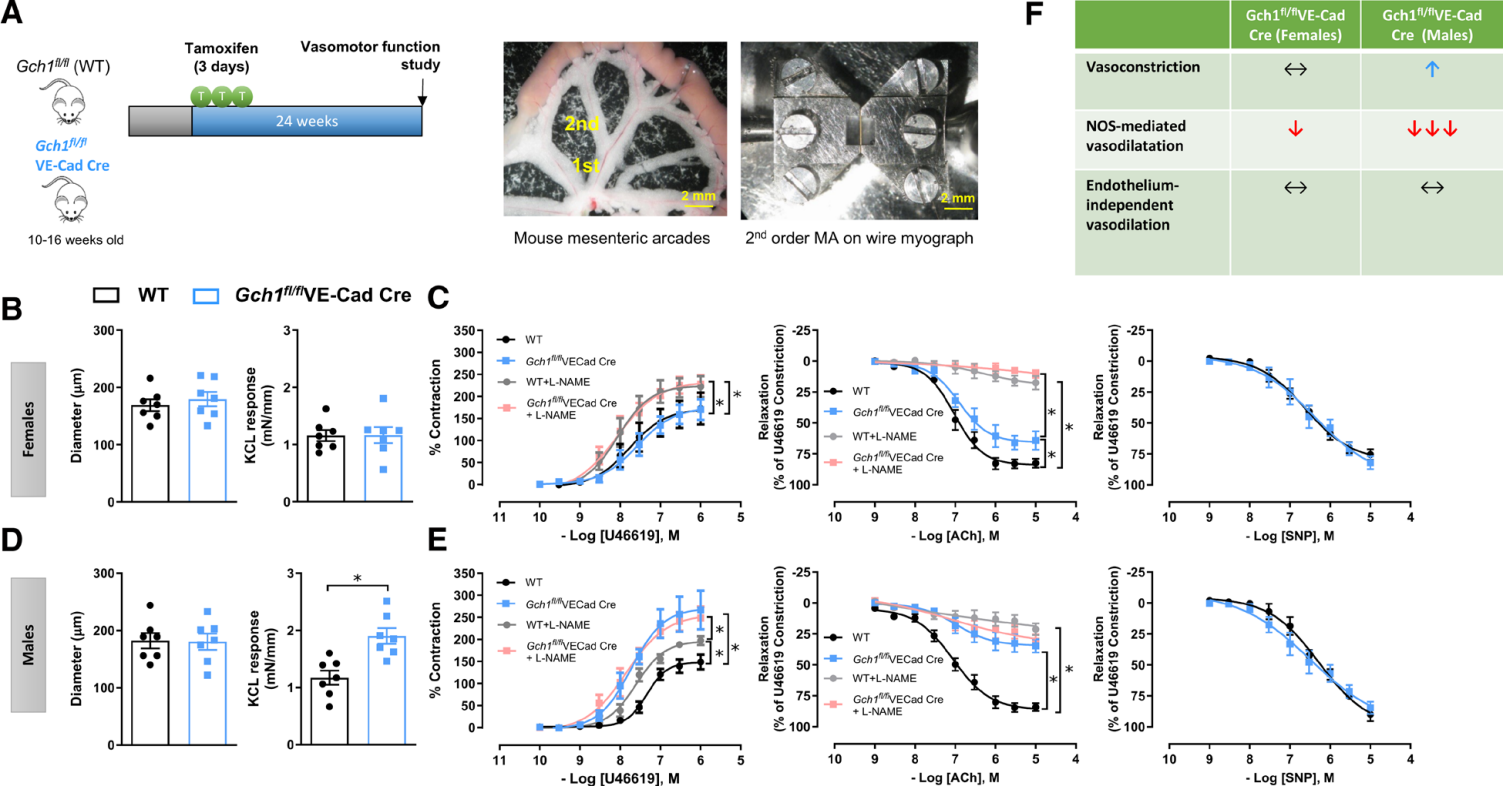
**Induction of endothelial cell *Gch1* deletion impairs resistance vessel function in a sex-specific manner. A**, (**Left**) Endothelial cell-specific *Gch1* knockout mice were generated through tamoxifen administration (2 mg/day for 3 consecutive days) starting at 10 to 16 weeks of age. Tissues were harvested at 24 weeks post-Cre-mediated *Gch1* deletion to assess vasomotor function. (**Right**) Representative image of a small intestine segment with associated mesenteric vasculature, highlighting first- and second-order branches of the mesenteric artery. Isometric tension studies were performed on second-order mesenteric arteries isolated from male and female mice using a wire myograph to assess vessel reactivity (**right**). **B** and **D**, Baseline diameter and maximal contractile response to 45 mmol/L KCl were measured in mesenteric arteries from female and male mice, respectively (*P<0*.05*; n=7 animals per group). **C** and **E**, Receptor-mediated vasoconstriction was induced using the thromboxane A₂ receptor agonist U46619. Responses were expressed as a percentage of the maximal KCl-induced constriction. Experiments were conducted in the presence or absence of the nonselective nitric oxide synthase (NOS) inhibitor L-NAME (Nω-nitro-l-arginine methyl ester; *P<0.05; n=7 animals per group). Endothelium-dependent vasodilatation in response to acetylcholine (ACh) was moderately impaired in mesenteric arteries from female *Gch1*^*fl/fl*^VE-Cad-Cre mice compared with wild-type controls (*P<0*.05*; n=7 animals per group). In contrast, male *Gch1*^*fl/fl*^VE-Cad-Cre mice exhibited severely blunted ACh-induced vasodilatation. Endothelium-independent vasodilatation in response to sodium nitroprusside (SNP), a nitric oxide donor, was comparable between genotypes in both females and males (n=7 animals per group). **F**, Summary of vasomotor function in resistance mesenteric arteries from *Gch1*^*fl/fl*^VE-Cad-Cre mice. Data are presented as mean±SEM.

To evaluate the long-term effects of endothelial *Gch1* deletion and BH4 deficiency on vascular function, vasomotor responses in resistance mesenteric arteries were assessed in WT and *Gch1*^*fl/fl*^VE-Cad-Cre mice 24 weeks after tamoxifen administration (Figure 4A). Wire myography was used to measure isometric tension responses.

In female mice, the diameters of second-order mesenteric arteries, as determined by the length-tension relationship, were comparable between WT and *Gch1*^*fl/fl*^VE-Cad-Cre mice (Figure 4B). Similarly, the vasoconstriction responses to 45 mmol/L KCl (Figure 4B) and the receptor-mediated vasoconstriction in response to the thromboxane A_2_ receptor agonist U46619 were comparable between *Gch1*^*fl/fl*^VE-Cad-Cre and WT female mice (Figure 4C). In the presence of the NOS inhibitor L-NAME, vasoconstriction to U46619 increased in both groups but remained comparable between the genotypes (Figure 4C). However, endothelium-dependent vasodilation in response to acetylcholine (ACh) was mildly impaired in female *Gch1*^*fl/fl*^VE-Cad-Cre mesenteric arteries (Figure 4C). This impairment was abolished in the presence of L-NAME, suggesting that the observed dysfunction was attributable to a loss of NOS-derived vasodilators (Figure 4C). Endothelium-independent vasodilation in response to the nitric oxide donor SNP remained comparable between genotypes (Figure 4C), indicating that the impairment in vasodilation was not due to altered vascular smooth muscle sensitivity to exogenous NO.

In male mice, the diameters of second-order mesenteric arteries were comparable between genotypes, as observed in female mice (Figure 4D). However, vasoconstriction in response to KCl was significantly enhanced in *Gch1*^*fl/fl*^VE-Cad-Cre mesenteric arteries from male mice compared with WT controls (Figure 4D). In contrast to the findings in females, receptor-mediated vasoconstriction to U46619 was also significantly increased in male *Gch1*^*fl/fl*^VE-Cad-Cre mesenteric arteries (Figure 4E). In the presence of L-NAME, vasoconstriction to U46619 was significantly enhanced in WT mesenteric arteries but remained unchanged in *Gch1*^*fl/fl*^VE-Cad-Cre mesenteric arteries, suggesting a complete loss of NO-mediated vascular tone in male *Gch1*^*fl/fl*^VE-Cad-Cre mice (Figure 4E).

Furthermore, endothelium-dependent vasodilation in response to ACh was severely impaired in *Gch1*^*fl/fl*^VE-Cad-Cre males (Figure 4E). In contrast to females, L-NAME had no effect on endothelium-dependent vasodilatation in male *Gch1*^*fl/fl*^VE-Cad-Cre arteries, while L-NAME completely abolished vasodilation in WT arteries (Figure 4E). This indicates a complete loss of NOS-derived vasodilatory function in BH4-deficient ECs in male mice. Nevertheless, similar to female mice, endothelium-independent vasodilation to SNP in males remained comparable between genotypes (Figure 4E).

These findings indicate that induction of endothelial *Gch1* deletion and BH4 deficiency leads to abnormal vascular function in the resistance mesenteric arteries of both male and female mice (Figure 4F). However, the striking impairment of endothelial function in male mice demonstrates sex-specific effects of BH4 loss that are not mitigated by adaptive or compensatory mechanisms.

### Induction of Maternal Endothelial *Gch1* Deletion and BH4 Deficiency Immediately before Pregnancy Causes Progressive Hypertension, Vascular Dysfunction, and Fetal Growth Restriction

**Figure 5.**
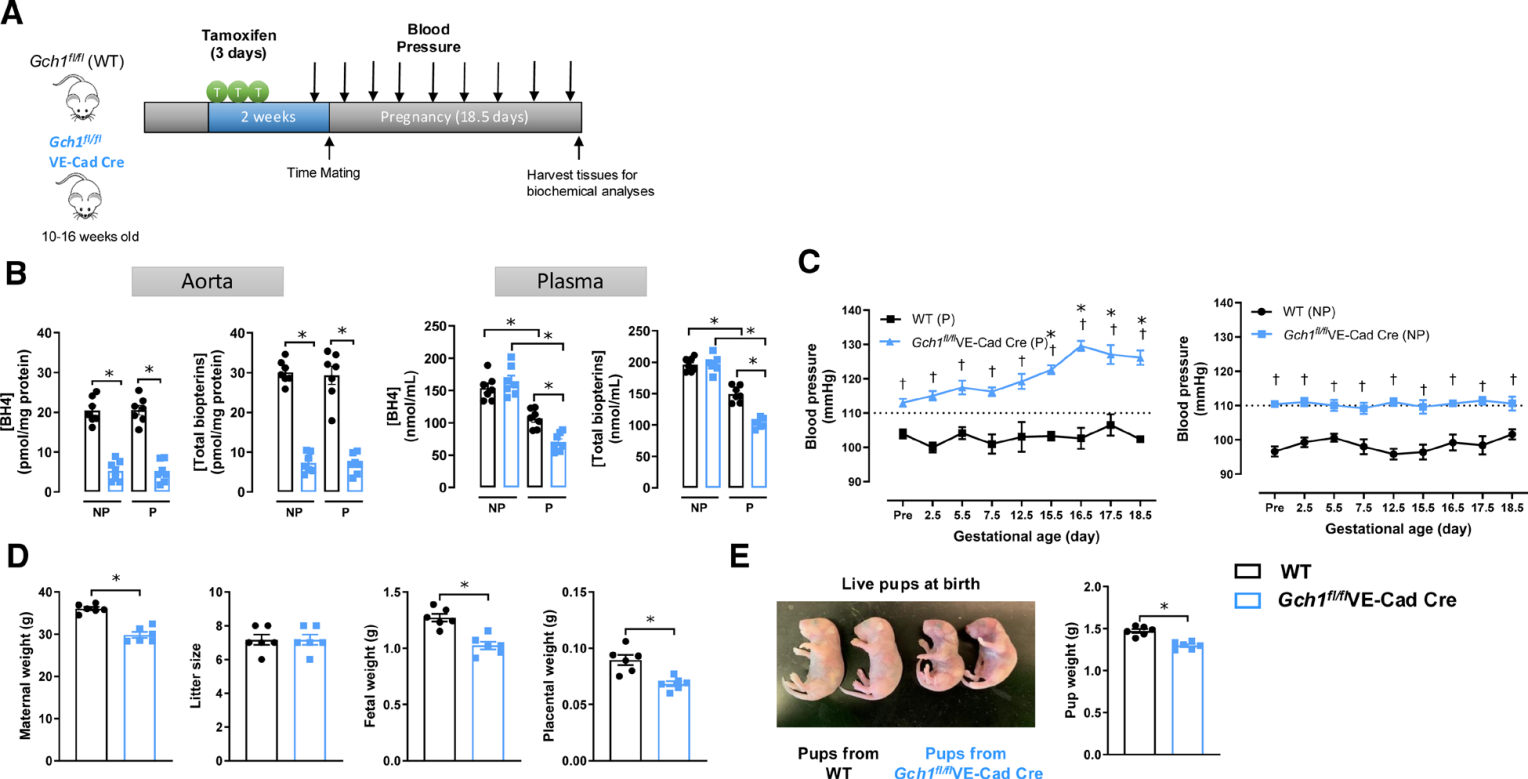
**Induction of maternal endothelial *Gch1* deletion and tetrahydrobiopterin (BH4) deficiency immediately before pregnancy causes progressive hypertension and fetal growth restriction. A**, Experimental protocol illustrating the inducible *Gch1* knockout strategy. Adult *Gch1*^*fl/fl*^ (wild-type [WT]) and *Gch1*^*fl/fl*^VE-Cad-Cre littermates were treated with tamoxifen (2 mg/day for 3 consecutive days) to activate Cre recombinase. Pregnancy was initiated by mating virgin female WT and *Gch1*^*fl/fl*^VE-Cad-Cre mice (ages 10–16 weeks) with male WT mice. Tissues were collected at embryonic day 18.5 (E18.5) of gestation or from nonpregnant control mice for subsequent biochemical and functional analyses. **B**, Biopterin levels were measured in aortas and plasma of nonpregnant (NP) and pregnant (P; E18.5) WT and *Gch1*^*fl/fl*^VE-Cad-Cre mice using high-performance liquid chromatography (HPLC; *P<0*.05*; n=7 animals per group). **C**, Blood pressure was measured by tail-cuff plethysmography in both WT and *Gch1*^*fl/fl*^VE-Cad-Cre mice before and during pregnancy (†*P*<0*.05*, vs genotypes; *P<0*.05*, vs *Gch1*^*fl/fl*^VE-Cad-Cre at baseline; n=7 animals per group; **left**). Tail-cuff BP in age-matched nonpregnant WT and *Gch1*^*fl/fl*^VE-Cad-Cre mice (†*P*<0*.05*, vs genotypes; n=7 animals per group; **right**). **D**, At gestation day 18.5 (E18.5), maternal body weight was significantly lower in *Gch1*^*fl/fl*^VE-Cad-Cre mice compared with WT mice (*P<0*.05*; n=7 animals per group). There were no significant differences in the number of pups per litter at E18.5. Fetal and placental weights from pregnant *Gch1*^*fl/fl*^VE-Cad-Cre mice were significantly lower than those from wild-type mice (*P<0*.05*; n=7 litters per group). **E**, Offspring weights at birth were recorded for both WT and *Gch1*^*fl/fl*^VE-Cad-Cre mice, with weight data averaged per litter. Growth restriction persisted postnatally, with offspring from *Gch1*^*fl/fl*^VE-Cad-Cre mice exhibiting significantly lower birth weights compared with those from wild-type mice (*P<0*.05*; n=6 litters per group). Data are presented as mean±SEM.

To investigate the role of inducing maternal endothelial *Gch1* deletion and BH4 deficiency in pregnancy, virgin female *Gch1*^*fl/fl*^VE-Cad-Cre and WT (*Gch1*^*fl/fl*^) mice (aged 10–16 years weeks) received tamoxifen and were then mated with male *Gch1*^*fl/fl*^ mice 2 weeks later (Figure 5A). This strategy ensures that embryos are not exposed to tamoxifen, so do not have endothelial *Gch1* deletion or BH4 deficiency, to test the specific effects of inducing maternal endothelial BH4 deficiency.

Aortic BH4 and total biopterin levels were significantly lower in both nonpregnant and pregnant *Gch1*^*fl/fl*^VE-Cad-Cre mice compared with their respective WT controls (Figure 5B). Plasma BH4 and total biopterin levels were also significantly reduced in pregnant mice of both genotypes, with a greater reduction observed in *Gch1*^*fl/fl*^VE-Cad-Cre mice (Figure 5B).

Consistent with earlier findings, tail-cuff BP was mildly elevated (≈5–10 mm Hg) in nonpregnant *Gch1*^*fl/fl*^VE-Cad-Cre mice compared with nonpregnant WT mice 2 weeks post-tamoxifen administration (Figure 5C). By gestation day 15.5 (E15.5), tail-cuff BP in pregnant *Gch1*^*fl/fl*^VE-Cad-Cre mice was significantly elevated above baseline levels, with a further increase observed at E18.5 compared with pregnant WT mice (Figure 5C). In contrast, BP remained unchanged in age-matched nonpregnant WT and *Gch1*^*fl/fl*^VE-Cad-Cre mice (Figure 5C). At gestation day 18.5 (E18.5), maternal body weight was significantly lower in *Gch1*^*fl/fl*^VE-Cad-Cre mice compared with WT mice (Figure 5D; WT: 36.0±1.0 g versus *Gch1*^*fl/fl*^VE-Cad-Cre: 29.8±1.7 g). There were no significant differences in the number of pups per litter at E18.5 or at birth between genotypes (Figure 5D). However, fetal and placental weights from pregnant *Gch1*^*fl/fl*^VE-Cad-Cre mice were significantly lower than those from WT mice (Figure 5D). This growth restriction persisted postnatally, with offspring from *Gch1*^*fl/fl*^VE-Cad-Cre mice exhibiting significantly lower birth weights compared with those from WT mice (Figure 5E; WT: 1.47±0.06 g versus *Gch1*^*fl/fl*^VE-Cad-Cre: 1.29±0.04 g).

**Figure 6.**
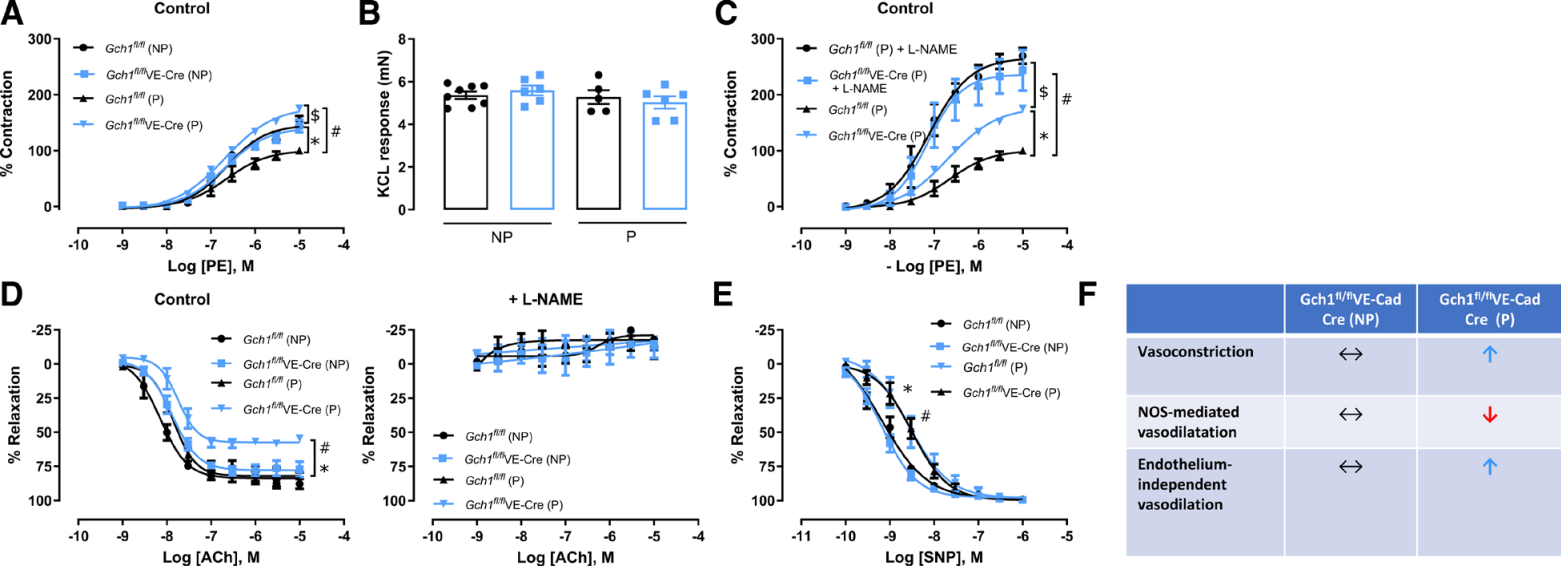
Induction of maternal endothelial *Gch1* deletion and tetrahydrobiopterin (BH4) deficiency immediately before pregnancy causes vascular endothelial dysfunction. Vascular function of aortic rings isolated from nonpregnant (NP) and pregnant (P) mice from both genotypes (*Gch1*^*fl/fl*^VE-Cad-Cre and wild-type [WT] littermates) at E18.5 was determined using wire myography. **A**, Percentage contraction in response to phenylephrine (PE) in NP and P mice from both genotypes (*#P*<0*.05*; WT (P) vs *Gch1*^*fl/fl*^VE-Cad-Cre (P); *P<0*.05*; WT (NP) vs WT (P); $*<0.05*; *Gch1*^*fl/fl*^VE-Cad-Cre (NP) vs *Gch1*^*fl/fl*^VE-Cad-Cre (P); n=5–7 animals per group). **B**, Vasoconstriction in response to KCL (60 mmol/L) was comparable between NP and P aortas from both genotypes. **C**, Percentage contraction in response to PE in pregnant aortic rings in the presence or absence of a nonselective nitric oxide synthase (NOS) inhibitor, L-NAME (Nω-nitro-l-arginine methyl ester; *P<0*.05*; WT (P) vs *Gch1*^*fl/fl*^VE-Cad-Cre (P); *#P*<0*.05*; WT (P) vs WT (P)+L-NAME; $*P*<0.05; *Gch1*^*fl/fl*^VE-Cad-Cre (P) vs *Gch1*^*fl/fl*^VE-Cad-Cre (P)+L-NAME; n=5–7 animals per group). **D**, Endothelium-dependent vasodilatation to acetylcholine (Ach) in aortic rings from NP and P mice from both genotypes the presence or absence of L-NAME (*#P*<0*.05*; WT (P) vs *Gch1*^*fl/fl*^VE-Cad-Cre (P); *P<0*.05*; *Gch1*^*fl/fl*^VE-Cad-Cre (NP) vs *Gch1*^*fl/fl*^VE-Cad-Cre (P); n=5–7 animals per group). **E**, Endothelium-independent vasodilatation in response to the nitric oxide donor, sodium nitroprusside (SNP; *#P*<0*.05*; WT (NP) vs WT (P); *P<0*.05*; *Gch1*^*fl/fl*^VE-Cad-Cre (NP) vs *Gch1*^*fl/fl*^VE-Cad-Cre (P); n=5–7 animals per group). **F**, Summary of vasomotor function in conduit vessels from NP and P *Gch1*^*fl/fl*^VE-Cad-Cre mice. Data are presented as mean±SEM.

To investigate the impact of maternal endothelial cell BH4 deficiency on vascular function, we assessed conduit arteries from both nonpregnant and pregnant *Gch1*^*fl/fl*^VE-Cad-Cre and WT mice using wire myography. Isometric tension studies of isolated aortas revealed no significant differences in phenylephrine-induced vasoconstriction between genotypes in the nonpregnant state (Figure 6A). However, in WT mice, vasoconstriction in response to phenylephrine was significantly reduced in pregnant aortas compared with nonpregnant aortas (Figure 6A), suggesting an increase in basal vasodilator-mediated vascular tone during normal pregnancy. In contrast, this pregnancy-associated reduction in phenylephrine-induced vasoconstriction was absent in *Gch1*^*fl/fl*^VE-Cad-Cre mice. Instead, vasoconstriction was significantly enhanced in pregnant *Gch1*^*fl/fl*^VE-Cad-Cre aortas compared with their nonpregnant counterparts (Figure 6A), indicating dysregulated vascular tone in the absence of endothelial BH4. This effect was not attributable to structural differences, as vasoconstriction to KCl remained similar across all groups (Figure 6B). In the presence of L-NAME, vasoconstriction in response to phenylephrine was markedly enhanced in both pregnant WT and *Gch1*^*fl/fl*^VE-Cad-Cre mice, abolishing genotype-dependent differences (Figure 6C). These findings suggest that the increased vasoconstrictor response observed in pregnant *Gch1*^*fl/fl*^VE-Cad-Cre mice is mediated by a loss of tonic eNOS-derived vasodilators.

In nonpregnant mice, there was a trend toward reduced endothelium-dependent vasodilation in response to ACh in *Gch1*^*fl/fl*^VE-Cad-Cre aortas compared with WT aortas; however, this difference did not reach statistical significance (Figure 6D). In pregnant mice, ACh-induced endothelium-dependent vasodilation was significantly impaired in *Gch1*^*fl/fl*^VE-Cad-Cre aortas compared with both pregnant WT and nonpregnant *Gch1*^*fl/fl*^VE-Cad-Cre mice (Figure 6D), indicating progressive endothelial dysfunction during pregnancy in the absence of endothelial BH4. Polyethylene glycol-conjugated catalase (polyethylene glycol-conjugated [PEG]-catalase), a hydrogen peroxide scavenger, did not significantly affect acetylcholine-induced vasodilatation in aortas isolated from *Gch1*^*fl/fl*^VE-Cad-Cre mice under nonpregnant conditions (Figure S4). In contrast, in aortic rings from pregnant *Gch1*^*fl/fl*^VE-Cad-Cre mice, PEG-catalase significantly reduced endothelium-dependent vasodilatation (Figure S4), indicating that hydrogen peroxide (H₂O₂) serves as an important compensatory mediator during pregnancy when endothelial BH4 is limited.

In the presence of L-NAME, ACh-induced vasodilation was completely abolished across all groups, confirming that eNOS is the primary mediator of endothelium-dependent vasodilation in both pregnant and nonpregnant mice. Furthermore, this finding demonstrates a loss of NOS-derived vasodilation in pregnant *Gch1*^*fl/fl*^VE-Cad-Cre aortas (Figure 6D). Pregnancy significantly increased the potency of endothelium-independent vasodilation to the nitric oxide donor SNP in aortas from both WT and *Gch1*^*fl/fl*^VE-Cad-Cre mice compared with nonpregnant WT mice (Figure 6E), suggesting that pregnancy enhances nitric oxide signaling in conduit arteries. These findings indicate that induction of maternal endothelial BH4 deficiency leads to abnormal vascular function during pregnancy (Figure 6F).

Taken together, these findings demonstrate that maternal endothelial-specific deletion of *Gch1*, immediately before pregnancy, leads to progressive pregnancy-induced hypertension, vascular and placental dysfunction, causing fetal growth restriction, highlighting the critical role of endothelial BH4 in maintaining maternal and uteroplacental vascular homeostasis during gestation.

## Discussion

This study provides additional new insights into the importance of endothelial *Gch1* and BH4 in regulating vascular function, BP, and pregnancy outcomes. Using a new VE-Cad-CreERT2 model, we studied the consequence of inducing BH4 deficiency in adult mice, eliminating developmental adaptations that limit the interpretation of the findings in constitutive Tie2cre models.^7,9,10^ This approach enables greater specificity in determining endothelial cell BH4 effects, and tests the immediate effects of loss of endothelial BH4 in adult mice, including immediately before pregnancy, providing new mechanistic insights into the role of BH4 in vascular homeostasis, and the potential for therapeutic targeting of BH4 effects in acquired cardiovascular disease states. The key findings are as follows: (1) Induction of endothelial BH4 deficiency in adult mice leads to marked hypertension and vascular dysfunction, with a much more striking phenotype than that observed in models of constitutive endothelial BH4 deficiency, identifying a requirement for endothelial cell BH4 in adult mice that is not mitigated by developmental or other adaptive effects, and highlighting BH4 as a targetable mechanism in acquired vascular disease states. Notably, the inducible model lacks the compensatory upregulation of eNOS protein and the H₂O₂-mediated vasodilation seen in constitutive models, reinforcing the pathophysiological relevance of BH4 deficiency in adult vascular disease contexts. (2) Induction of endothelial BH4 deficiency in adult males causes progressive hypertension, whereas females develop a milder and nonprogressive increase in BP, highlighting sex-specific differences in vascular responses. (3) Male *Gch1*^*fl/fl*^VE-Cad-Cre mice exhibit severe NO-mediated vasodilatory impairment, while females retain partial endothelial function via compensatory vasodilatory mechanisms in resistance arteries. (4) In pregnant females, endothelial BH4 deficiency causes gestational hypertension and fetal growth restriction, accompanied by vascular dysfunction and impaired uteroplacental function. Since offspring were not exposed to tamoxifen, and therefore did not have endothelial *Gch1* deletion, these adverse pregnancy outcomes are specifically related to maternal effects of inducing endothelial cell BH4 deficiency.

Previous studies using constitutive endothelial BH4-deficient mice (*Gch1*^*fl/fl*^Tie2cre) have shown a mild but significant elevation in BP (≈7 mm Hg), without major differences between male and female mice.^3^ The present study extends these findings by discovering a more striking effect of inducible endothelial-specific *Gch1* deletion in adult mice using the VE-Cadherin (VE-Cad)-CreERT2 system. In contrast to the mild elevation of BP previously reported in *Gch1*^*fl/fl*^Tie2cre mice with constitutive *Gch1* deletion, we observed a much greater, progressive and sex-specific elevation in BP over 24 weeks following inducible *Gch1* deletion. Notably, male *Gch1*^*fl/fl*^VE-Cad-Cre mice exhibited a progressive increase in BP, whereas female mice developed a milder, nonprogressive hypertensive response that stabilized early after induction.

These observations suggest that developmental or other adaptive mechanisms modulate the pathophysiologic effects of constitutive endothelial BH4 deficiency in the *Gch1*^*fl/fl*^Tie2cre mouse, and these differ between males and females. Sex differences in endothelial function and hypertension are well-documented, with estrogen playing a protective role by enhancing NO signaling and reducing oxidative stress.^16^ Critically, the present study highlights more striking consequences of endothelial cell BH4 deficiency when induced in adult animals, highlighting the potential of BH4-mediated effects as a major therapeutic opportunity in acquired cardiovascular disease states where BH4 deficiency develops as a contributor to disease pathogenesis.

Endothelial BH4 plays a critical role in maintaining eNOS function and vascular tone, particularly in resistance arteries that regulate systemic BP. In the constitutive *Gch1*^*fl/fl*^Tie2cre model, where BH4 deficiency is present from early development, the endothelium appears to adapt by activating compensatory mechanisms such as H₂O₂-mediated vasodilation and endothelium-derived hyperpolarization-type responses.^7,8^ These alternative pathways help preserve endothelial function despite reduced NO availability, suggesting that early and sustained BH4 deficiency allows for long-term vascular adaptation.

Our data demonstrate that PEG-catalase had no effect on acetylcholine-induced vasodilatation in aortas from nonpregnant *Gch1*^*fl/fl*^VE-Cad-Cre mice, suggesting that unlike the constitutive model, H₂O₂ is not an effective compensatory mediator under baseline conditions. However, during pregnancy, PEG-catalase significantly attenuated vasodilatation in *Gch1*^*fl/fl*^VE-Cad-Cre, indicating that H₂O₂ becomes functionally important as a compensatory mechanism only in the pregnant state. This distinction provides novel mechanistic insight into how timing and physiological context influence vascular adaptation to BH4 deficiency.

In contrast, the inducible *Gch1*^*fl/fl*^VE-Cad-Cre model, in which BH4 is depleted acutely in adulthood, these protective mechanisms fail to provide adaptation, particularly in male mice, resulting in marked eNOS uncoupling, reduced NO bioavailability, impaired vasodilation, and progressive hypertension. These findings highlight a potentially much greater importance of endothelial cell BH4 availability as a factor in acquired vascular disease states, such as hypertension, than previously suggested by constitutive endothelial cell BH4 deficiency models. Furthermore, this conclusion indicates that targeting BH4 effects may have much greater therapeutic potential in acquired rather than constitutive vascular disease states.

Normal pregnancy relies on healthy endothelial function, that mediates reduced systemic vascular resistance due to enhanced endothelial NO bioavailability, and growth of the placental vasculature, supporting the hemodynamic adaptations that are essential for fetal development.^3,5,17,18^ Globally important pregnancy complications such as preeclampsia and fetal growth restriction are characterized by endothelial dysfunction, with a casual role for BH4.^3^ In female *Gch1*^*fl/fl*^VE-Cad-Cre mice, induction of endothelial cell BH4 deficiency immediately before pregnancy, when the animals remain normotensive, results in a progressive increase in BP during gestation. This result demonstrates that the adverse effects of endothelial BH4 deficiency in pregnancy are not dependent upon preexisting hypertension, nor are they prevented by adaptive effects that act to mitigate the hypertensive response to induction of endothelial BH4 deficiency in nonpregnant female mice, compared with male mice. The absence of endothelial *Gch1* deletion in offspring, which are conceived after the effects of tamoxifen administration, reinforces the role of maternal, rather than fetal, endothelial cell BH4 deficiency in pregnancy-induced hypertension and fetal growth restriction.

Furthermore, immunoblot analysis of ECs isolated from *Gch1*^*fl/fl*^VE-Cad-Cre mice confirmed marked loss of GTPCH protein, without changes in eNOS protein. This contrasts with previous observations in constitutive models where eNOS levels were upregulated, likely due to chronic oxidative stress and H₂O₂ signaling. This finding underscores the limited adaptive response following acute BH4 depletion, and further highlights differences between constitutive and inducible models. Consistent with this, there were no genotype-dependent differences in phospho-eNOS at Serine 1177 or Threonine 495 when normalized to total eNOS. Thus, the endothelial dysfunction observed in this study is unlikely to reflect altered eNOS phosphorylation stoichiometry and is instead consistent with BH4 deficiency-driven eNOS uncoupling rather than changes in upstream kinase/phosphatase signaling.

Pregnant *Gch1*^*fl/fl*^VE-Cad-Cre mice exhibited marked endothelial dysfunction, characterized by exaggerated vasoconstriction responses normalized by NOS inhibition, confirming the loss of tonic eNOS-derived vasodilation. Endothelium-dependent vasodilation was significantly impaired, with enhanced endothelium-independent vasodilation to SNP, suggesting a compensatory increase in vascular smooth muscle sensitivity to NO. However, this adaptation was insufficient to prevent pregnancy-induced hypertension and fetal growth restriction. These findings highlight the therapeutic potential of targeting endothelial BH4 effects in hypertensive disorders of pregnancy such as preeclampsia, and improving fetal outcomes.^19^

Critically, there is translational relevance to these findings. In human studies, endothelial BH4 deficiency has been implicated in preeclampsia and essential hypertension, with reduced BH4 levels and eNOS uncoupling observed in placental vessels and systemic arteries. The inducible *Gch1*^*fl/fl*^VE-Cad-Cre model, therefore provides a clinically relevant platform to study acquired endothelial dysfunction, mimicking disease pathogenesis more closely than constitutive models.

Taken together, this study establishes the first inducible, endothelial-specific model of BH4 deficiency in adult mice, that reveals the direct effect of acquired endothelial BH4 deficiency, without the potentially mitigating effects of developmental, hematopoietic or other adaptive mechanisms. The findings reveal striking, sex-specific effects of inducing endothelial BH4 deficiency in adult animals, causing increased and progressive hypertension compared with the mild BP phenotype observed in models of constitutive endothelial BH4 deficiency. Furthermore, onset of endothelial BH4 deficiency before pregnancy in female mice causes pregnancy-induced hypertension and fetal growth restriction, independent of fetal genetic effects. These findings provide critical new insights into the requirement for BH4 in endothelial function in adults, the importance of sex-dependent vascular adaptations, and pregnancy-related complications. Together, these findings demonstrate that the VE-Cadherin inducible model is a powerful and translationally relevant system for studying acquired vascular disease, and that targeting BH4 could have significant therapeutic implications in both chronic cardiovascular conditions and hypertensive pregnancy syndromes.

## Perspectives

This study demonstrates that acute, adult-onset endothelial BH4 deficiency leads to striking and sex-specific vascular dysfunction and hypertension, with more severe outcomes than previously reported in constitutive knockout models. These findings have important implications for our understanding of acquired vascular disease, highlighting that endothelial BH4 availability is not only essential during development but remains a critical determinant of vascular homeostasis in adulthood. The observation that inducible endothelial *Gch1* deletion produces progressive hypertension in males and pregnancy-induced hypertension with fetal growth restriction in females underscores the context-dependent vulnerability of the endothelium to BH4 deficiency.

Importantly, these results point to a failure of adaptive mechanisms in the adult vasculature to compensate for acute BH4 loss, contrasting with the partial compensation observed in developmental models. This suggests that therapeutic strategies aimed at preserving or restoring endothelial BH4 levels may be especially effective in adult-onset cardiovascular conditions, including essential hypertension and preeclampsia.

Furthermore, the inducible VE-Cad-Cre model offers a powerful platform to dissect endothelial-specific contributions to complex disease states and to test therapeutic interventions targeting endothelial redox balance, eNOS coupling, or BH4 metabolism. These insights collectively position endothelial BH4 as a viable target for precision medicine approaches in cardiovascular and pregnancy-related disorders.

## ARTICLE INFORMATION

### Sources of Funding

This study was supported by a British Heart Foundation (BHF) Program Grants (RG/F/22/110085), BHF Project Grant (PG/19/48/34433), BHF Chair award (CH/16/1/32013), Oxford BHF Centre of Research Excellence (RE/18/3/34214).

### Disclosures

None.

## Supplementary Material


