# Supplementary material for: Inducible Endothelial Gch1 Deletion Reveals Rapid, Sex-Specific Effects on Blood Pressure and Pregnancy Outcomes
Source: Hypertension. 2025 Oct 1;83(2):e25058. doi: 10.1161/HYPERTENSIONAHA.125.25058 (PMC12822761; doi:10.1161/HYPERTENSIONAHA.125.25058)
Supplement: Supplementary file 1 [file hyp-83-e25058-s001.pdf]

Inducible Endothelial *Gch1* Deletion Reveals Rapid, Sex-Specific Effects on Blood Pressure and Pregnancy Outcomes

Running Title: *Inducible endothelial BH4 deficiency, Hypertension, and Pregnancy*

Surawee Chuaiphichai[1*], Desson Au-Yeung[1], Christopher A.R. Whiteman[1], Sarah L. Cook[1], Eileen McNeill[1], Gillian Douglas[1], Keith M. Channon[1]

[1]Division of Cardiovascular Medicine, British Heart Foundation Centre of Research Excellence, Radcliffe Department of Medicine, University of Oxford, Oxford, OX3 9DU, UK

[*]Corresponding authors:

Surawee Chuaiphichai, DPhil
Division of Cardiovascular Medicine
British Heart Foundation Centre of Research Excellence,
Radcliffe Department of Medicine,
University of Oxford,
Oxford, OX3 9DU, UK
Tel: +44(0)1865 287662
e-mail: surawee.chuaiphichai@cardiov.ox.ac.uk

Supplementary Figure S1

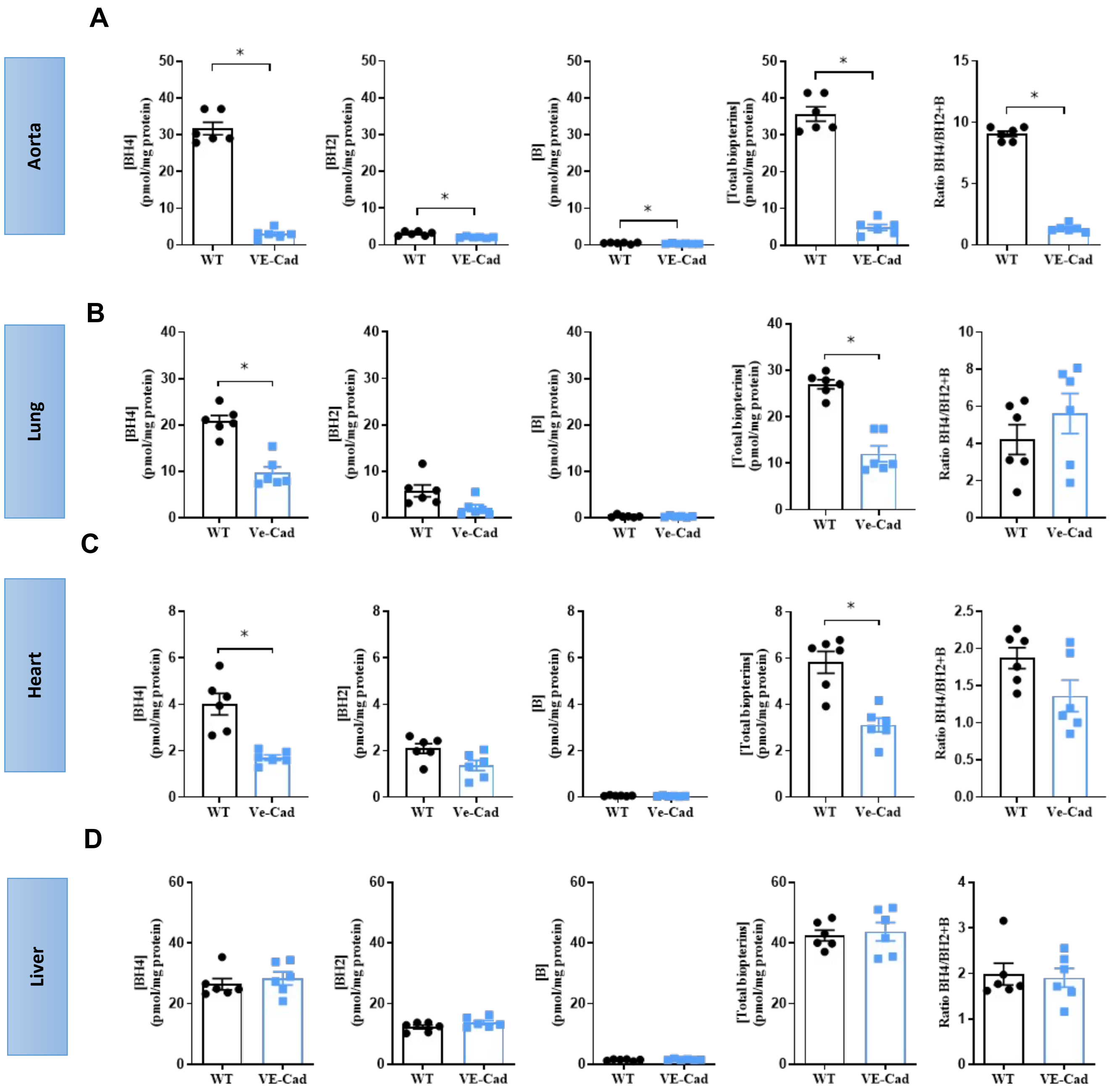

Figure S1. Endothelial Cell-Specific Loss of *Gch1* Leads to Endothelial Cell BH4 Deficiency in Endothelial Cell-Rich Tissues, but Not in Non-Endothelial Cell-Rich Tissues. Adult $Gch1^{fl/fl}$ (wild-type, WT) and $Gch1^{fl/fl}$VE-Cad-Cre littermates (10-16 weeks old) were administered tamoxifen (2 mg per day for 3 consecutive days). Tissues were harvested 2 weeks post-tamoxifen administration. Intracellular concentrations of BH4 and oxidised biopterins (BH2 and total biopterins (B)) were measured in A) aortas, B) lungs, C) hearts, and D) livers from $Gch1^{fl/fl}$ (wild-type, WT) and $Gch1^{fl/fl}$VE-Cad-Cre littermates using high-performance liquid chromatography (HPLC) with electrochemical and fluorescence detection, respectively ($*P < 0.05$, n = 6 animals per group). Data are presented as mean ± SEM.

Supplementary Figure S2

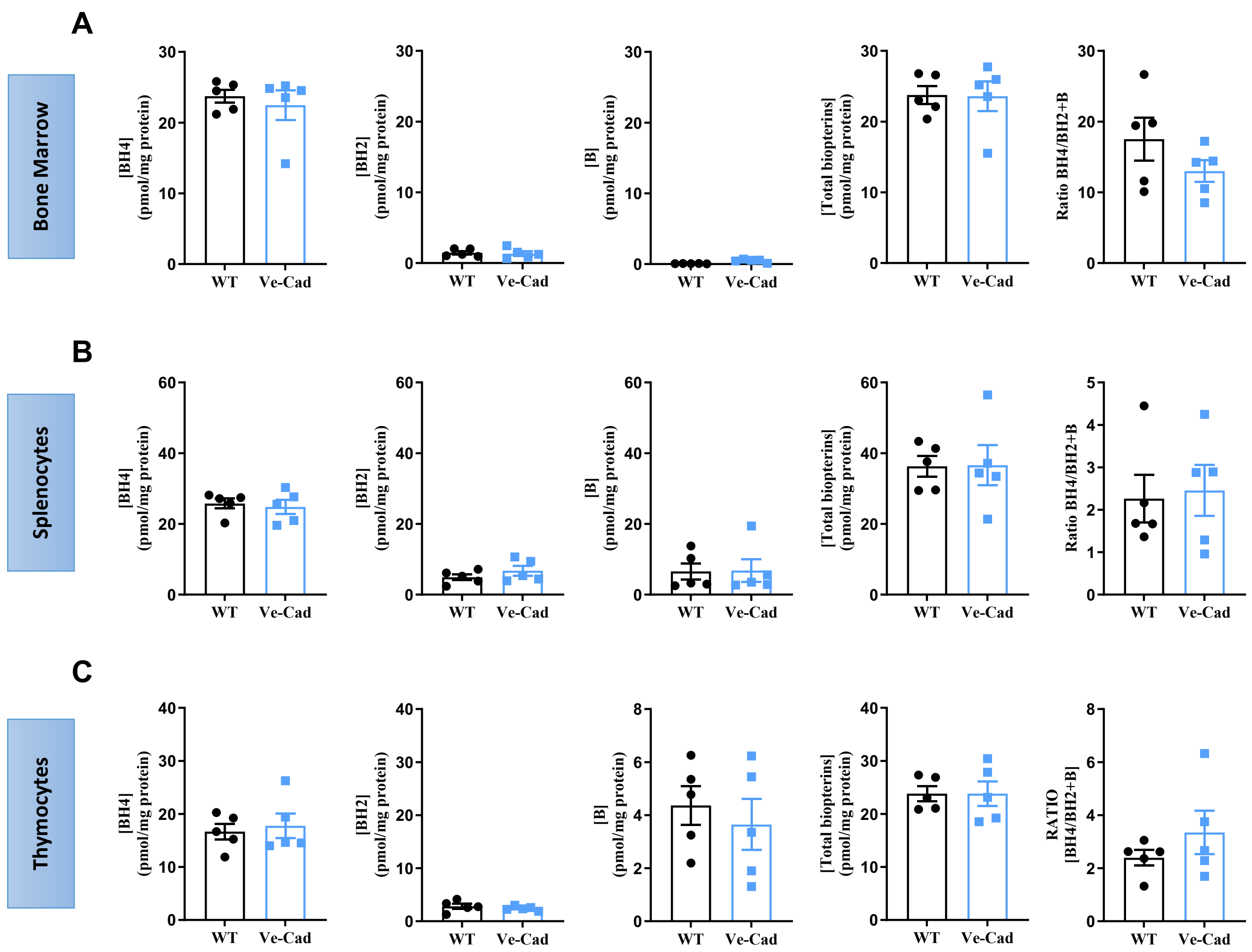

Figure S2. Endothelial Cell-Specific Loss of *Gch1* does not alter BH4 levels in haematopoietic cells. To induce the conditional deletion of *Gch1*, adult *Gch1*$^{fl/fl}$ (wild-type, WT) and *Gch1*$^{fl/fl}$VE-Cad-Cre littermates (10-16 weeks old) were administered (2 mg per day of tamoxifen for 3 consecutive days). Tissues were harvested 4 weeks post tamoxifen administration. Intracellular concentrations of BH4 and oxidised biopterins (BH2 and total biopterins (B)) were measured in A) bone marrow, B) splenocytes, and C) thymocytes from *Gch1*$^{fl/fl}$ (wild-type, WT) and *Gch1*$^{fl/fl}$VE-Cad-Cre littermates using high-performance liquid chromatography (HPLC) with electrochemical and fluorescence detection, respectively (n = 5 animals per group). Data are presented as mean ± SEM.

Supplementary Figure S3

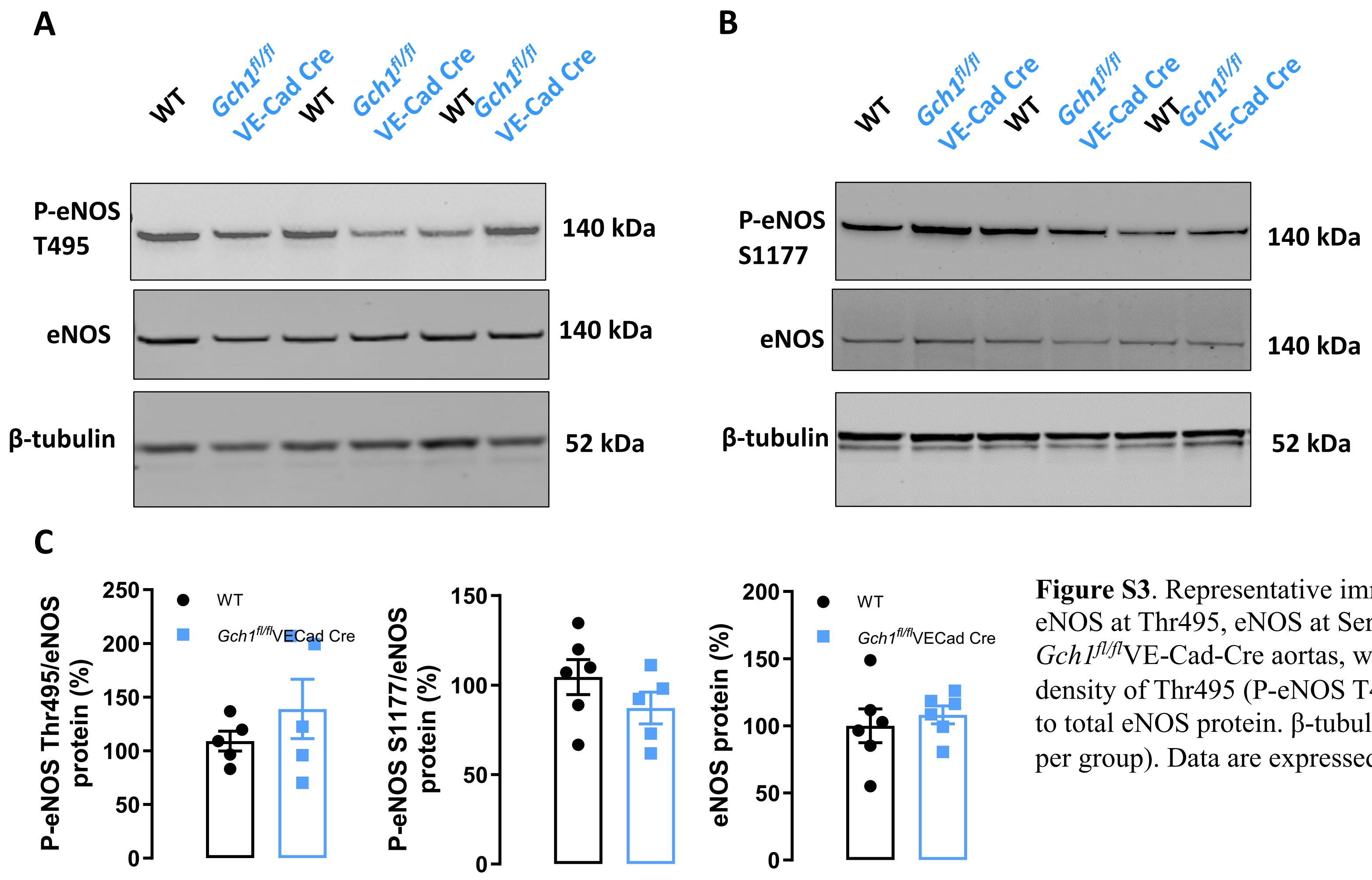

Figure S3. Representative immunoblots showing **(A and B)** Phosphorylation of eNOS at Thr495, eNOS at Ser1177 and total eNOS protein in wild-type (WT) and *Gch1*$^{fl/fl}$VE-Cad-Cre aortas, with (**C**) quantitative data, measured as percentage band density of Thr495 (P-eNOS T495) to total eNOS protein, Ser1177 (P-eNOS S1177) to total eNOS protein. β-tubulin was used as loading control. below. (*n* = 5-6 animals per group). Data are expressed as mean ± SEM.

Supplementary Figure S4

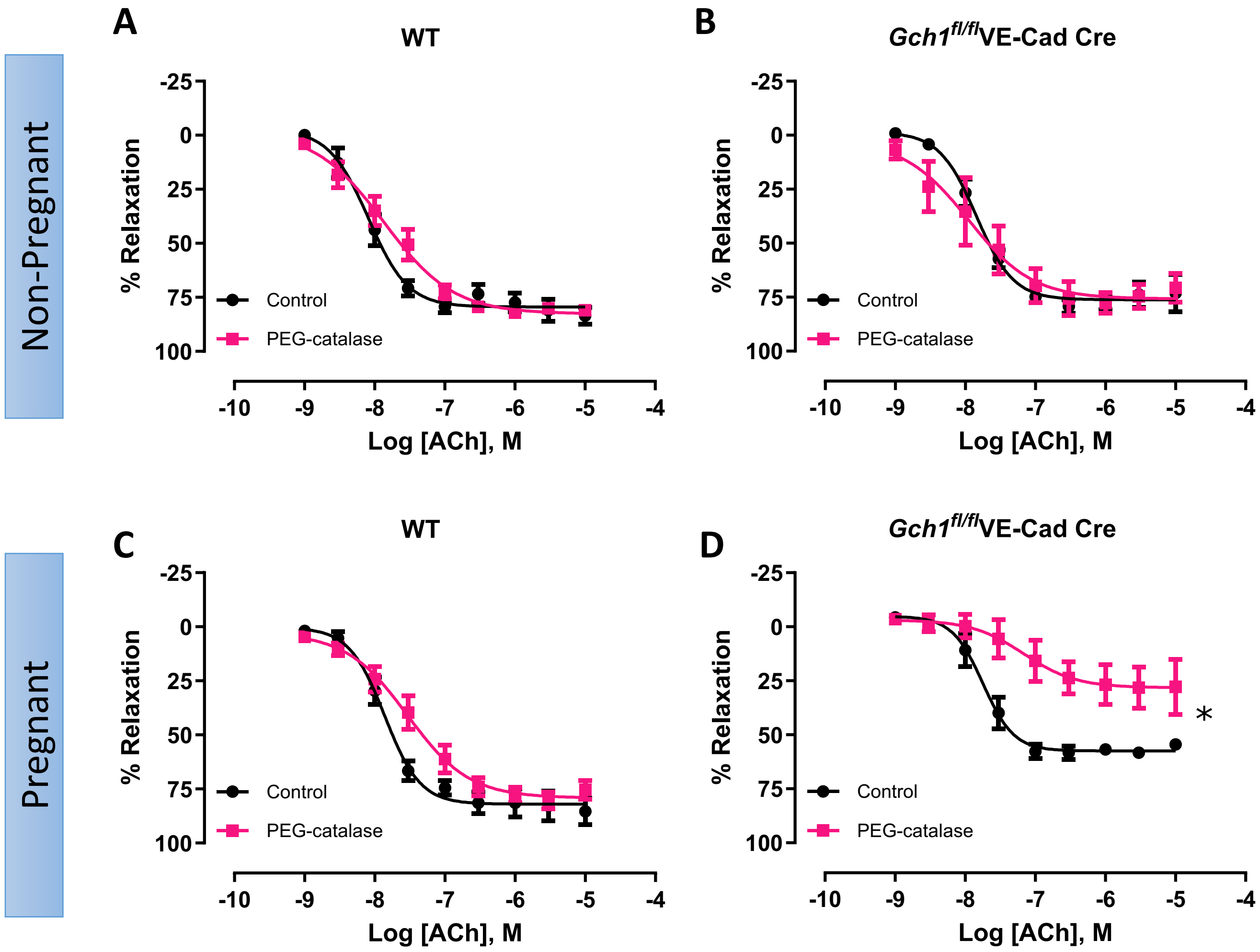

Figure S4. Contribution of H_2O_2-mediated vasodilatation in mouse aortas from non-pregnant (A) WT and (B) *Gch1*$^{fl/fl}$VE-Cad-Cre mice and pregnant (C) WT and (C) *Gch1*$^{fl/fl}$VE-Cad-Cre mice. Endothelium-dependent vasodilatation to acetylcholine (Ach) was determined in the presence and absence of PEG-catalase (400 unit/ml). *$P < 0.05$; significantly different as indicated; n = 5 to 8 animals per group. Data are expressed as mean ± SEM
